# Method to allocate voting resources with unequal ballots and/or education

**DOI:** 10.1016/j.mex.2020.100872

**Published:** 2020-03-20

**Authors:** Theodore T. Allen, Muer Yang, Shijie Huang, Olivia K. Hernandez

**Affiliations:** a Integrated Systems Engineering, The Ohio State University, Columbus, OH 43210, United States; b Opus College of Business, University of St. Thomas, Saint Paul, MN 55105, United States; c Advanced Analytics, Nationwide E&S, Scottsdale, AZ 85258, United States

**Keywords:** Simulation optimization, Multiple comparisons, Comparison with the best, Election systems, Voting machine, Allocation, Resource management, Consumer requirements management, Indifference-zone

## Abstract

Apportionment in election systems refers to determination of the number of voting resources (poll books, poll workers, or voting machines) needed to ensure that all voters can expect to wait no longer than an appropriate amount, even the voter who waits the longest. Apportionment is a common problem for election officials and legislatures. A related problem is “allocation,” which relates to the deployment of an existing number of resources so that the longest expected wait is held to an appropritate amount. Provisioning and allocation are difficult because the numbers of expected voters, the ballot lengths, and the education levels of voters may all differ significantly from precinct-to-precinct in a county. Consider that predicting the waiting time of the voter who waits the longest generally requires discrete event simulation.

• The methods here rigorously guarantee that all voters expect to wait a prescribed time with a bounded probability, e.g., everyone expects to wait less than thirty minutes with probability greater than 95%.
• The methods here can handle both a single type of resource (e.g., voting machines or scan machines) and multiple resource types (e.g., voting machines and poll books).
• The methods are provided in a freely available, easy-to-use Excel software program.

## Graphical abstract

**Figure:**
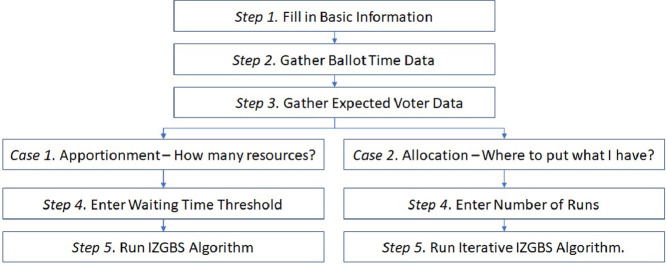

Specifications Table

**Table:** 

Subject Area:	Engineering
More specific subject area:	*Operations Research & Simulation Optimization*
Method name:	*Service Time Estimation and Indifference-Zone Generalized Binary Search*
Name and reference of original method:	*Nowak, R. D. (2011). The Geometry of Generalized Binary Search. IEEE Transactions on Information Theory 57(12), 7893–7906.*
Resource availability:	http://www.blying.com/sitebuildercontent/sitebuilderfiles/voteizgbs.alphav7ready.xlsm http://www.blying.com/sitebuildercontent/sitebuilderfiles/iiepomsfranklincountryelection_analysis_datanomacro.xlsx

## Method details

Apportionment and allocating voting resources are important problems for our society. Two of us have served as expert witnesses on U.S. court cases in which political partisans have (we believe) inflicted waiting lines differentially on voters of other parties and likely changed the results in multiple races. Also, we developed multiple methods to provision and allocate voting machines [5,12,13]. However, none of these previous methods have included features provided by the discrete event simulations described here (accounts for finite election days, predictable rush periods, and multiple types of resources) and rigorous guarantees.

In our linked publication [6], we prove that the Indifference-Zone Generalized Binary Search (IZGBS) method provides the needed solution quality guarantees and describe our free enabling software. Here, we describe our enabling software in greater detail together with information relating to how to use the methods and how to interpret the related outputs. Our linked publication extends the Generalized Binary Search (GBS) in Nowak [10]. The GBS can guarantee termination in a small number (logarithmically bounded) of steps with an optimal solution but not if the evaluations are only within an indifference-zone of being correct with known probability. This indifference-zone is associated with simulation or other empirical evaluations. This explains the need for the IZGBS. It also explains why the methods are guaranteed only to provide solutions within an indifference parameter of a target with a bounded probability, i.e., there is a high chance that all can expect to wait less than a desired amount. For example, that everyone will expect to wait less than 30.5 min with the parameter 0.5 min.

The proposed method presented here covers both Case 1 (Apportionment) and Case 2 (Allocation). Both methods share the first three steps. To preview, the steps are:

Step 1. Fill in Basic Information
Step 2. Gather Ballot Time Data
Step 3. Gather Expected Voter Data
  Case 1. Apportionment
Step 4. Enter Waiting Time Threshold
Step 5. Run IZGBS Algorithm
  Case 2. Allocation
Step 4. Enter Number of Runs
Step 5. Run Iterative IZGBS Algorithm.

The notation that we use is as follows:

q= the number of locations for allocations (could be precincts or vote centers).
N= the number of available machines (if there is a finite number).
$R_{i}=$ the number of available voters in location *i*, i.e., the number registered minus those who voted early.
$T_{i}=$ the predicted turnout fraction of the available voters in location *i.* This could be overall fraction *T*_0_.
$E_{i}=R_{i}\times T_{i}$ the expected number of voters who will vote in location *i*.
$r_{t}=$ the arrival rate ratio for hour *t* of the day, e.g., the rate is double for first two hours and last two hours.
$\lambda_{i,t}=$ the arrival rate at location *i* in hour *t*.
$\gamma_{i}=$ the number of extra issues at location *i* (or another measure of length) with shortest and longest, *γ_s_* and *γ_l_*.
$\left(a_{s},b_{s},c_{s}\right),\;\left(a_{l},b_{l},c_{l}\right)=$ the shortest and longest ballot triangular parameters (minimum, maximum, mode).
$n_{0}=$ the initial sample size or number of replicates in the simulation.
$z_{1-p}=$ the (1−p)th percentile of the normal distribution.
$x_{i}=$ the combination of resources at location *i* which could be a vector of two types of resources.
$\mu_{0}=$ the threshold of quality, e.g., that everyone should expect to wait thirty minutes or less.
δ= the indifference amount relating for the quality threshold, e.g., being less than δ=1 min.
$\mu (x,\lambda_{i,t},\gamma_{i}\;\forall i,t)=$ the true mean service time as it depends on the number of machines *x, λ*_*i, t*_, *γ_i_* ∀*i, t*.
$\hat{\mu }\left(x,\lambda_{i,t},\gamma_{i}\;\forall i,t\right)=$ the estimated true mean service time as it depends on the number of machines *x, λ*_*i, t*_, *γ_i_* ∀*i, t*.

*H*_j_ the hypotheses about whether *μ*(*x, λ*_*i, t*_, *γ_i_* ∀*i, t*) ≤ *μ*_0_ that have not been ruled out at iteration *j* of the algorithm.

With this notation, the approach steps vary from inputting numbers describing the election to collecting data to applying a rigorous method.

*Step 1. (Basic Information) Go through the steps on the “Home” sheet*

Typically, we are focusing on the bottleneck resource type which might be the number of Direct Recording Electronic (DRE) voting machines or voting booths. At a minimum we select one resource type, although we might want optimally to select two types of resources, e.g., poll book and DRE or poll worker and voting booth combinations. Then, we select the maximum or quantile-based waiting time as the objective. With our methods and simulation, the choice has limited (if any) affect on solution quality, and is up to user preferences. The maximum waiting time objective specifies the expected waiting time of the longest waiting voter will be less than a pre-set threshold (*μ*_0_), e.g., 80 min. Another possible way of expressing goals is in terms of percentiles. For example, the 99% percentile voter might experience a wait less than the preset threshold.

The indifference parameter (*δ*) is the value greater than the threshold (*μ*_0_) that might be acceptable. The threshold could be 80 min and the indifference parameter might be 0.5 min. Then, everyone will expect to wait less than 80.5 min if the “Max” objective is selected. The probability, initial sample size, and batch size relate to specific guarantees of the method, the number of simulated batches and number of replicates in each batch. A typical Election Day starts one hour before the doors open.

*Step 2. (Gather Ballot Time Data) Gather 20 or more times from the shortest and longest ballot*

Next, we need to estimate the service times for all locations. In our work on *Phillip Randolph* vs*. Ruth Johnson* (federal case #2:16-cv-11844, [4]) we measured average service times in two Michigan locations: 4.0 min in Ontwa and 15.27 min in Detroit. This occurred in part because of ballot length differences and/or voter education levels and/or the level of care given. Yet, failing to address such disparities in allocation will predictably lead to discrimination as we testified in that case. Unless there is a clear pattern as in Detroit, we suggest loading the shortest and longest ballots into the system and timing twenty voters on each. This can likely be done through ensuring enough distance in early voting that voter's rights will not be infringed. The times needed are the times from which the resource is free until it is free again. With twenty times, the user can formally fit or simply estimate the shortest likely time, the most likely time, and the longest likely time. This is done for both the shortest and longest ballots and entered in the software together with the corresponding measure of ballot length (*γ_i_*), e.g., the number of extra issues (e.g., 10 local issues) after the mandatory politician races (e.g. typically 24 races in a major Franklin County, Ohio election). If data is not available for ballots, they can be elicited from experts such as voting officials using a questionnaire approach possibly similar to the elicitation in Allen and Maybin [2]. Using linear interpolation, the general distribution for location *i* is then:

(1) $$triangular(a_{s}+\frac{\left(a_{l}-a_{s}\right)\times \left(\gamma_{i}-\gamma_{s}\right)}{\gamma_{l}-\gamma_{s}},b_{s}+\frac{\left(b_{l}-b_{s}\right)\times \left(\gamma_{i}-\gamma_{s}\right)}{\gamma_{l}-\gamma_{s}},\;c_{s}+\frac{\left(c_{l}-c_{s}\right)\times \left(\gamma_{i}-\gamma_{s}\right)}{\gamma_{l}-\gamma_{s}}\;\text{for}\;i=1,\ldots ,q.$$

*Step 3. Enter the number of expected or likely voters and the number registered for each location*

The closer to Election Day the planning is done, the more exact the forecasts can be because early voters are not eligible to vote twice, of course. Also, information about the level of public interest and excitement surrounding the election could conceivably be used. We have investigated multiple forecasting procedures. Working with election officials or experts could produce a principled forecast for each location. Using a fraction ($T_{i}=T_{0}$) of eligible voters with a small allotment for risk seems to be in keeping with current practices. These numbers are entered for each location (*E_i_*) with the measure of ballot length, e.g., the number of extra issues in the “Single Resource” or “Multiple Resource” sheets for one and two resource types respectively. Then, the day is broken into arrival periods indexed by *t* in the “Home” page. The dimensionless period relative rates are entered as *r_t_* (relative to each other). For example, the rush period at the beginning and end of the day might have $r_{t}=2$. Then, the estimated period average arrival rate is (*λ*_*i, t*_):

(2) $$\lambda_{i,t}=\frac{E_{i}\times r_{t}}{\frac{60\mathrm{mins}}{\mathrm{hr}}\times \sum_{i}^{h}r_{i}}=\frac{R_{i}\times T_{i}\times r_{t}}{\frac{60\mathrm{mins}}{\mathrm{hr}}\times \sum_{i}^{h}r_{i}}\text{for}\forall \;t\text{and}i.$$

The arrivals are assumed to be non-stationary Poisson (the usual uncoordinated assumption, e.g., [1] p. 40). The remaining steps depend on the user case, i.e., apportionment (case 1) or allocation (case 2).

*Case 1. “Apportionment” – Determining the number of needed resources to meet a given performance measure*

*Step 4. Set the allowable threshold* (*μ*_0_) *for the performance measure*

In Step 1, the performance measure was selected, e.g., the mean time of the voter who waits the longest. In this step, the user determines a performance requirement threshold for that measure. For example, the user might select $\mu_{0}=80\;\text{min}$ as a reasonable value for the voter who waits the longest. Note that we are evaluating locations individually so that the longest-waiting voter time across locations can exceed this value by some amount. We are simply constraining the expected waiting time on an average day for that location. With 500 or more locations, one location could have its worst day out of 1000 days. Therefore, while applying 80 min as a threshold may lead to a high seeming apportionment, it could also lead to expected waits at some location of as much as 100 min or more at one of the locations. We expect those effects will generally be minor even if one location is above the threshold, due to multiplicity.

*Step 5. Run the Indifference*-*Zone Generalized Binary Search (IZGBS) to determine the number of resources needed*

Our companion paper describes IZGBS and provides rigorous guarantees of its solution quality. The algorithm is presented there in relation to generic hypotheses which could apply to any resource constrained simulation optimization problem. For a single resource, we simply perform a binary search each time using simulation and a statistical comparison, e.g., t-testing to prove that the mean is above or below the threshold with an indifference parameter. In other words, we can accept an incorrect simulation-based comparison if the true mean is within *δ* of being feasible. Note if we say the threshold is 30 min and the indifference parameter is 0.5, we are guaranteeing a limit of 30.5 min in this scheme. Here, we focus the notation on election system apportionment with simulation or empirical-based estimates, $\hat{\mu }\left(x,\lambda_{i,t},\gamma_{i}\;\forall i,t\right)$. The Indifference-Zone Generalized Binary Search (IZGBS) algorithm is as follows.

**(IZGBS) Initialize**
The set of hypotheses, *H*_0_, possibly accounting for the more-is-better assumption.
**Loop Until** {One hypothesis remains}
Select{*x* to evaluate such that the most hypotheses will be eliminated regardless
  of the comparison results $\hat{\mu }\left(x,\lambda_{i,t},\gamma_{i}\;\forall i,t\right)>\mu_{0}$ or $\hat{\mu }\left(x,\lambda_{i,t},\gamma_{i}\;\forall i,t\right)\leq \mu_{0}$}
Evaluate $\hat{\mu }\left(x,\lambda_{i,t},\gamma_{i}\;\forall i,t\right)>\mu_{0}$ or $\hat{\mu }\left(x,\lambda_{i,t},\gamma_{i}\;\forall i,t\right)\leq \mu_{0}$ (expensive simulation or experimental step)
**End Loop**

For a single resource, the selection phase involves picking the approximate halfway point on the relevant interval. For example, searching 1 to 100, we try 50, then 25, then 13, then 19, then 16, then 15, and terminate. In this example, we might conclude that 16 resources are enough but 15 are too few. With multiple resource types, the hypotheses are more complicated. Next, we consider the case of finite resources and try to derive the minimum possible threshold performance value.

*Case 2. You currently have a fixed resource number and want to assign them to locations ("Allocation").*

Step 4. Run approximate allocation and simulate to find a reasonable solution.

It may be desirable to allocate existing resources approximately and quickly. In our numerical examples the method in this section requires only a few seconds. The method here uses a fast approximation for the average waiting time based on strong assumptions. The assumptions considered here include that the queues are in steady state, i.e., election day is imagined to be extremely long so that long term averages apply. Also, the arrivals follow a Poisson distribution and the services or voting times are exponentially distributed. This defines the so-called “M/M/c” queue ([1] p. 81). Using an approximation to the M/M/c queue from Kolesar and Green [7], a formula for the needed number of machines can be applied. We used a heuristic formula for the delay probability which is exp(−90/q). Then, a simple binary search to equitably apply all the available machines is as follows. In the software, this is called the fast and approximate method.

**Fast Approximate Kolesar and Green Allocation**
Initialize the threshold j=0, $\mu_{H}=300\;\text{min}$, $\mu_{L}=1\;\text{min}$.
**Loop**
n=0; $q=0.5(\mu_{H}+\mu_{L})$
  **For**{i=1,…,q} (loop over locations)
   $n_{i}=\lambda_{i,1}/\mu_{i,1}-z_{e^{-90/q}}\sqrt{\lambda_{i,1}/\mu_{i,1}}$
   $n=n+n_{i}$
  End For
  If{*n* < *N*} Then {$\mu_{L}=\;\mu_{j}$}
  Else {$\mu_{H}=\;\mu_{j}$}
**End Loop When {**|n−N|<10**}**

The fast and approximate method will give reasonable solutions which account for variable ballot lengths or variable education levels. Yet, there are no rigorous guarantees for the solution quality based on simulation. For example, it cannot be guaranteed that all voters can expect to wait less than thirty or even eighty minutes.

*Step 5. (Optional) Slow but rigorous method to guarantee equitable allocation*

It may be desirable to allocate resources with a rigorous guarantee that all voters can expect to wait less than a predetermined amount with a bounded probability. The rigor of the method IZGBS method is established in a linked publication. The method here is adapted to the voting system context. Because this method involves repeated applications of a rigorous method, the final iteration offers a Bonferroni bounded guarantee that all voters will expect to wait less than the lowest possible threshold.

**(IZGBS Allocation) Initialize**
Initialize the threshold j=0, $\mu_{H}=300\;\text{min}$, $\mu_{L}=1\;\text{min}$.
**Loop**
n=0; j=j+1; $\mu_{j}=0.5\left(\mu_{H}+\mu_{L}\right)$
  **For**{i=1,…,q} (loop over locations)
  Apply IZGBS(*μ_j_, λ*_*i, t*_, *γ_i_* ∀*i, t*) generate *n_i_*
  $n=n+n_{i}$
  End For
  If{*n* < *N*} Then {$\mu_{L}=\;\mu_{j}$}
  Else {$\mu_{H}=\;\mu_{j}$}
**End Loop When {**$\mu_{j}-\mu_{j-1}<0.1\;\text{min}$**}**

We feel that this method offers the best available rigorous guarantee in which variable ballot lengths are addressed.

## Method validation

The software and methods have been studied in a court case analysis, i.e., *Philip Randolph* vs*. Ruth Johnson*. The method reduction to practice is described in the associated expert witness reports. The dataset for our method validation here is an assemblage of the information supplied by the Ohio Franklin County BOE. It is adapted from Huang [9]. It includes the number of registered voters, number of expected Election Day voters, and votes-weighted average number of issues on ballot by polling station for the 2012 Franklin County presidential election.

In the dataset, a total of 808,578 voters registered for the election and 71.1% voted. Also, 39.35% of the registered voters voted on Election Day across 475 polling stations. The number of Election Day voters varied significantly from 76 to 2976 by polling location, and the number of issues on ballot varied from 3 to 9. The wide variations across sites greatly complicates design of a system guaranteeing equal voting access. In Franklin County, polls are open for 13 h on Election Day, and the majority of the voting is completed on DRE machines. Poisson non-stationary arrival process with morning and evening surges is applied. In the software that we developed, users can customize the peaks and valleys of the arrival rates throughout the Election Day as shown in Table 1.

**Table 1:** The arrival rates considered in the method validation.

In Ohio, polls are open for 13 h from 6:30 a.m. to 7:30 p.m. From our past experience and data from individual DRE machines [13], we assume the voter arrival rate doubles for the first two hours (6:30 a.m. to 8:30 a.m.) and last two hours (5:30 p.m. to 7:30 p.m.) compared to the rest of the day with a base arrival rate. In addition, we employ an early voter arrival with the base arrival rate starting 5:30a.m.a.m. to preload the system. Then, for a polling station with 2976 expected voters, the base arrival rate (5:30a.m. to 6:30a.m., 8:30a.m. to 5:30p.m.) can be derived from Eq. (2) as $\lambda_{i,1}=2.76$, and the rush hour arrival rate is $\lambda_{i,t}=$ 5.51. We assume the above-mentioned arrival pattern is shared across all polling stations in the Franklin County.

Technically, the number of expected Election Day voters will not exceed the number of registered voters *R_i_*. Therefore, the Election Day voting system is modeled as a constrained non-stationary Poisson process, and the number of registered voters is applied as the maximum number of voters who could enter the system.

In a typical polling station, two services are usually considered. The first service is the check-in process carried out by poll workers, and the second service is the balloting process using the physical resources. For most of the time, the physical resources tend to be the bottleneck, but a potential cascading problem could be caused by inefficient planning of staff at check-in tables. To the best of our knowledge, there were some other voting machine allocation methods developed, as introduced in the next section, but they all focus solely on a single resource, and fail to account for the variation generated by the number of poll workers at the check-in table and its effect on the length of lines.

Observation data of the check-in process collected in the 2016 Franklin County Primary Election is used for the service time distribution modeling. A distribution *triangular*(0.9, 2.9, 1.1) is found with an average processing time of 1.63 min. The check-in time is assumed to be consistent across multiple polling stations, but the processing time a voter spends to mark and cast a ballot is expected to vary significantly by the ballot length. Allen and Bernshteyn [5] found the service time of balloting is highly correlated with the number of issues on the ballot, which played a critical role in the unequal treatment of voters in the previous Franklin County presidential elections. Data collected from a mock election study is used to model the variation of the balloting processing times across different locations.

Our mock study was designed specifically for ES&S DREs used in Franklin County with 60 carefully selected voters by balancing race, education level, and voter experience level. We find the service time for five issues on the ballot follows a distribution of *triangular*(3.7, 8.5, 6.0) with an average of 6.07 min, and the service time for eleven issues follows a distribution of *triangular*(4.6, 14.9, 9.0) with an average of 9.50 min. By applying a multiplier, the balloting time is constructed proportionally as a function of ballot length. Therefore, for location *i* with *γ_i_* number of issues, it will follow a distribution of $triangular(3.7+\frac{\left(4.6-3.7\right)\times \left(\gamma_{i}-5\right)}{11-5},\;8.5+\frac{\left(14.9-8.5\right)\times \left(\gamma_{i}-5\right)}{11-5},\;6.0+\frac{\left(9.0-6.0\right)\times \left(\gamma_{i}-5\right)}{11-5})$. Assume it takes an average of 0.75 min for voter and machine preparation, the time the voter stands in front of the machine is given in Table 2 by applying the aforementioned method. Approximately 0.57 min is added for every additional issue on the ballot.

**Table 2:** Balloting service time distribution by ballot length.

Ballot Length	Distribution (min)	Average (min)
3	triangular(4.15, 7.12, 5.75)	5.67
4	triangular(4.3, 8.18, 6.25)	6.24
5	triangular(4.45, 9.25, 6.75)	6.82
6	triangular(4.6, 10.32, 7.25)	7.39
7	triangular(4.75, 11.38, 7.75)	7.96
8	triangular(4.9, 12.45, 8.25)	8.53
9	triangular(5.05, 13.52, 8.75)	9.11
10	triangular(5.2, 14.58, 9.25)	9.68
11	triangular(5.35, 15.65, 9.75)	10.25

Next, we introduce the interfaces of the IZGBS election resource allocation software to facilitate the application of IZGBS method and provide the number of resources to be deployed to guarantee a certain service level. DES is integrated into the software to evaluate voter waiting times.

**Fig. 1:**
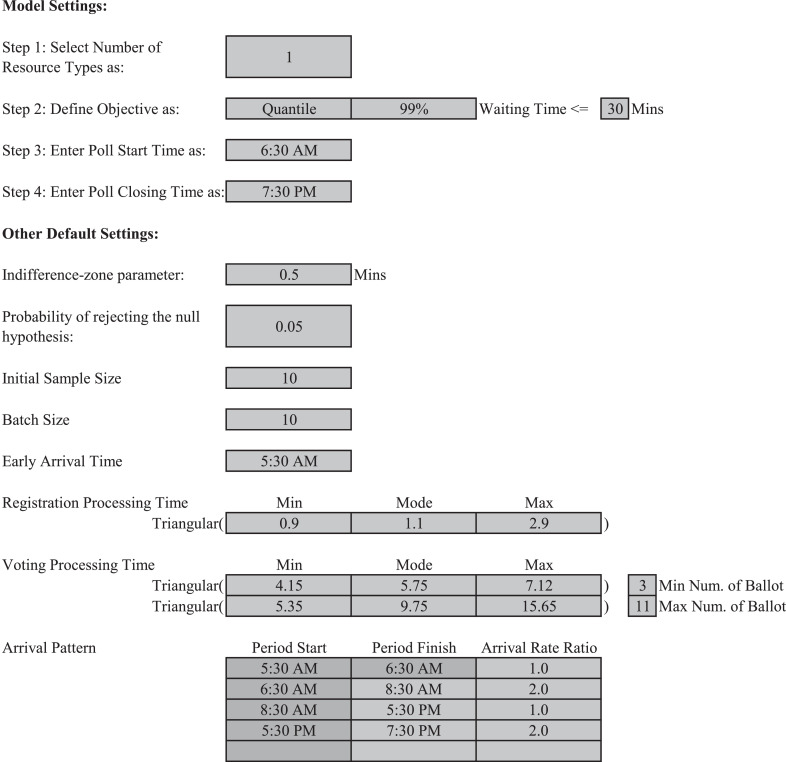
Parameter entry interface of IZGBS election resource allocation software.

The interfaces in Fig. 1 and Table 2 allow end-users to easily alter the design factors listed below regardless of their knowledge in IZGBS or DES:

• *Number of Resource Types:* User can select either 1 or 2 as the number of resource types considered. Typically, a voting system with a single resource type considers physical resource as the only bottleneck resource; while for a system with two resource types, both staff and physical resources are considered having limited capacities that could affect the voter flow.
• *Objective:* User can choose average, maximum or quantile-based waiting time as the performance metric (*μ*) to meet the service standard (*μ*_0_). For the Franklin County elections, we set *μ* as 0.99-quantile of the voter waiting time distribution, and *μ*_0_ as 30 min, according to the recommendation delivered by the Presidential Commission on Election Administration (2014).
• *Poll Start Time, Poll Closing Time and Early Arrival Time:* Poll start time indicates when the polling stations are open and start processing voters. Although voters could be pre-loaded in the system starting with the early arrival time, which is common in practice, their waiting time increment will not start before the poll opens. When a poll closes, new arrivals will be discontinued, but any voter waiting in the queue will still be served. For Franklin County, we set poll start time, closing time and early arrival time as 6:30 a.m., 7:30 p.m. and 5:30 a.m., respectively.
• *Alpha:* 1−α is the desired confidence level. We set *α* as 0.05, which indicates we have at least 95% confidence of selecting the correct hypothesis simultaneously for all steps.
• *Indifferencezone parameter:* This is the smallest difference (*δ*) that is critical to be detected by IZGBS. For Franklin, we set *δ* as 0.5 min, which means when the maximum waiting time $\mu \leq \mu_{0}-\delta =29.5$ mins, a feasible system declaration is expected. When $\mu \geq \mu_{0}+\delta =30.5$ min, the feasibility is rejected. When 29.5 min < *μ* < 30.5 min, feasible or infeasible declarations are both recognized as correct selection.
• *Initial Sample Size: n*_0_ is the number of observations used to estimate the variance in the first stage. The value $n_{0}=10$ is selected for the Franklin County case.
• *Batch Size:* For Franklin County, a batch size of 10 could guarantee the approximate data normality of waiting time at 0.99-quantile for all polling stations.
• The other settings for the registration service time, voting service time, arrival pattern, expected voters, registered voters and number of issues on ballot are discussed in Huang ([9], Chapter 4).

In this section, we review two alternative resources allocation approaches which are considered to be easy to implement and are commonly used in practice. Those two methods are also applied to the 2012 Franklin County Presidential Election data for validation and comparison considering a single resource and queue.

Queuing theory is a very important operations research domain to study waiting lines and resource allocations. It has been well studied and widely applied in many disciplines, including service process design [14]. The queuing formula considered in this section is developed by Kolesar and Green [7], and later adopted by Allen [1] to determine the number of voting machines needed in an election. KG method uses a normal approximation to Erlang's delay formula for a steady state M/M/s-type queuing system with Poisson arrivals and exponentially distributed inter-arrival. The service times are assumed to be IID according to an exponential distribution. Kolesar and Green [7] also suggested the application of KG method in a non-stationary environment.

According to the KG method, the smallest number of servers *c* needed to achieve a target probability of delay *p* can be calculated using the analytical queuing formula below:

(3) $$c=\lambda /\mu +z_{1-p}{\left[\lambda /\mu \right]}^{1/2}+1/2$$

where $z_{1-p}$ is the (1−p)th percentile of a standard normal distribution, and the target probability of delay *p* represents the desirable percentage of customers will experience any wait for the service.

For the 2012 Ohio Franklin County presidential election, we assume the voter arrival follows a Poisson distribution with $\lambda =\frac{R_{i}\times T_{i}}{13h\times \;60\text{min}/h}/\text{min}$ across the 13-h Election Day. The service rate *μ* can be calculated using the average service time implied by Table 3, which accounts for variable ballot length across polling stations. To determine the desirable percentage of customers with any service delays, we apply the same total number of machines derived by IZGBS. With a total of 3952 DRE machines allocated to 475 polling stations, we find $z_{1-p}=0.9977$ and p=0.1592. In other words, a total of 15.92% of voters will experience delay in the balloting process across the Franklin County. Applying $z_{1-p}=0.9977$ to each individual polling location, the results of machine allocation are listed in Table 5.

**Table 3:** Single resource interface of IZGBS election resource allocation software.

**Table 4:** Multiple resources interface of IZGBS election resource allocation software.

**Table 5:** Performance evaluation for election resources allocation strategies.

	IZGBS	PA	KG
Total Number of Machines Deployed	3952	4865	3952
Max of Avg Waiting Time (min)	8.0	23.0	16.0
Avg of Avg Waiting Time (min)	5.2	3.7	5.2
Std Dev of Avg Waiting Time (min)	1.4	3.9	3.0
Max of Max Waiting Time (min)	33.5	68.8	54.7
Avg of Max Waiting Time (min)	27.1	22.6	26.8
Std Dev of Max Waiting Time (min)	2.9	10.2	7.0
Max of 99th Quantile Waiting Time (min)	32.2	66.7	53.5
Avg of 99th Quantile Waiting Time (min)	25.5	20.9	25.2
Std Dev of 99th Quantile Waiting Time (min)	3.0	10.2	7.2
# Center with 99th Quantile wait >= 30.5 min	11	64	107
# Center with Max wait >= 30.5 min	57	76	135

Another method which is commonly used in practice by many election boards is to allocate voting resources in proportion to the number of registered voters at each polling station. This method is intuitive but it simply considers an identical turnout rate and service time across all locations. In Ohio, the Secretary of State for boards of elections mandated a minimum of one DRE voting machine per 175 registered voters for each county starting in 2013. This ratio is not universal, for example, New York State recommends one DRE device for every 550 registered voters excluding voters in inactive status. Here, we apply the 175 registered voter-to-DRE ratio to derive the number of machines as listed in Huang [9]. We find a total of 4865 machines are needed, compared to the 3952 machines derived by the IZGBS method applying a 30-minutes-or-less waiting time. In other words, if the BOE could allocate those 4865 machines scientifically, the expected maximum voter waiting time should be less than 30 min across Franklin County.

**Fig. 2:**
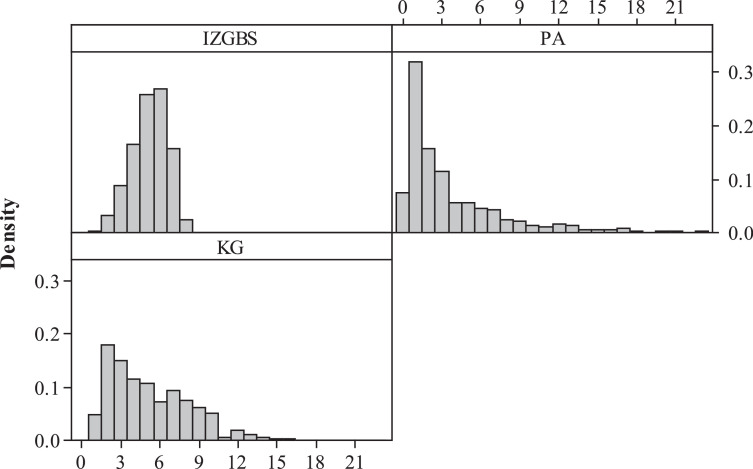
Performance evaluation of expected average waiting times

**Fig. 3:**
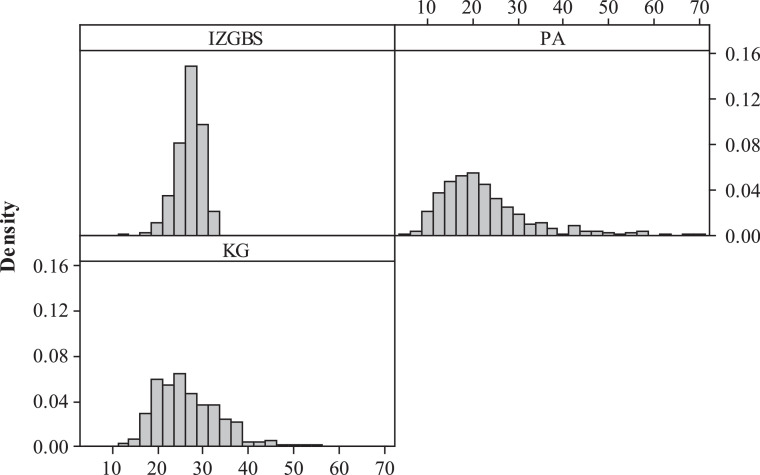
Performance evaluation of expected maximum waiting times

**Fig. 4:**
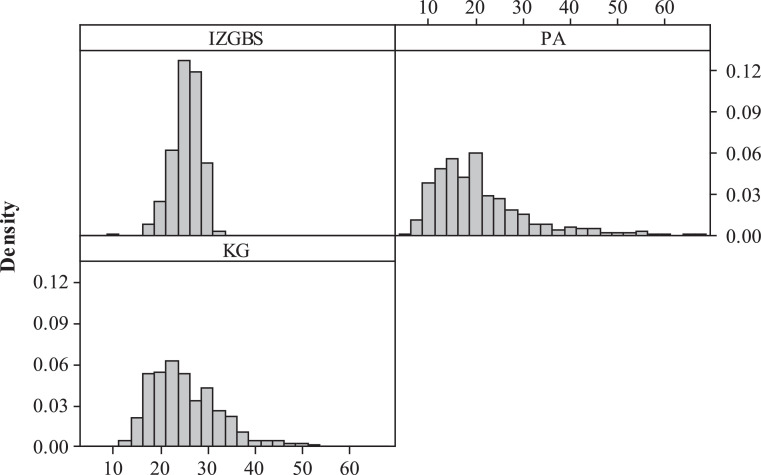
Performance evaluation of expected 99th quantile waiting times.

Next, we consider the scenario with voting machine being the only bottleneck resource in Franklin County to compare different machine allocation strategies. The aforementioned IZGBS software accommodates the constrained non-stationary voter arrivals, pre-loaded queues, and non-steady-state queues. KG, and Proportional Allocation (PA) which is allocation proportional to the number of voters, methods are also applied towards the same dataset for efficiency comparison. We apply high fidelity simulation with 100 replicates to compare the expected waits for those three allocation strategies using the 2012 Ohio Franklin County election data. The complete results are attached in Huang [9], with the key information summarized in Table 5, Fig. 2, Fig. 3, Fig. 4.

Generally, the IZGBS approach yields a total of 3952 voting machines, approximately 18.8% less compared to the traditional PA rule. As we expected, PA method delivers an overall lower waiting time, including “Avg of Avg”, “Avg of Max” and “Avg of Quantile”, due to the extra resources deployed. IZGBS significantly outperforms PA and KG at all the other performance metrics, including “Max of Avg Waiting Time”, “Std Dev of Avg Waiting Time”, “Max of Max Waiting Time”, “Std Dev of Max Waiting Time”, “Max of Quantile Waiting Time” and “Std Dev of Quantile Waiting Time”, although more resources are consumed by PA scenario.

By employing IZGBS and setting the objective of 99th quantile at 30 min, the “Max of Max Waiting Time” is determined as 33.5 min, compared with 68.8 and 54.7 min derived by the PA and KG methods, respectively. The standard deviation of maximum waiting time is efficiently reduced to 2.9, and this convergence really helps with the equity across different locations. To evaluate the accuracy of IZGBS algorithm, we look at the metric of “# Center with 99th Quantile wait >= 30.5 min”, only 2.3% of locations performs beyond the given service level plus *δ*, and the maximum waiting times across all polling stations are well controlled below 32.2 min.

Although PA and KG methods are simpler, they fail to consider the stochastic environment of non-steady-state queues and non-stationary voter arrival pattern. In addition, PA assumes the voter turnout rate and service time are identical across all polling stations. In contrast, the consideration of realistic complications provides IZGBS the basis of accuracy to achieve system efficiency and equity at the minimum cost. The converged waiting times across different locations significantly improve the equity and avoid discrimination against certain areas with long ballots and voters sharing similar demographic characteristics.

We also find the expected waits can be significantly changed by adding or removing a single resource. For example, for a location with 880 expected voters and 12 voting machines, the max waiting time can be increased from 27 min to 46 min by removing one single machine, which confirms the importance of guaranteeing the rigorousness of proposed procedure.

An in-depth discussion and additional details are in Huang [9]. Computational time may still be an issue. In future work, meta-modeling and multi-fidelity experimentation may be helpful for reducing times, e.g., see Huang and Allen [8]. Instead of running all location simulations as part of an overall optimization, metamodels can be developed from individual locations. Then, the metamodels may be used in place of some simulations in the optimization. Design of experiments methods can provide fast surrogates for simulation models (e.g., Allen and Bernstheyn [3] for linear models or [8], for Kriging models). Also, Woods, Schenk, and Allen [11] and Yang et al. [13] review resilience and related concepts which might be relevant for the design of precincts or voting locations that can recover quickly from long lines.

## CRediT authorship contribution statement

**Theodore T. Allen:** Conceptualization, Funding acquisition, Project administration. **Muer Yang:** Formal analysis, Methodology. **Shijie Huang:** Software, Data curation. **Olivia K. Hernandez:** Software, Writing - review & editing.

## Declaration of Competing Interest

The authors declare no conflict of interest.

## Appendix Supplementary materials

Image, application 1
