# Supplementary material for: Method to allocate voting resources with unequal ballots and/or education
Source: MethodsX. 2020 Mar 20;7:100872. doi: 10.1016/j.mex.2020.100872 (PMC7205765; doi:10.1016/j.mex.2020.100872)
Supplement: Supplementary file 1 [file mmc1.pdf]

Author Credit Statement

MethodX Method to Allocate Voting Resources with Unequal Ballots and/or Education

Theodore T. Allen Conceptualization; Funding acquisition; Project administration; Original draft
Muer Yang Formal analysis; Methodology
Shijie Huang Software; Data curation
Olivia K. Hernandez Writing - review & editing
